# Impact of Distance on Mode of Active Commuting in Chilean Children and Adolescents

**DOI:** 10.3390/ijerph14111334

**Published:** 2017-11-02

**Authors:** Fernando Rodríguez-Rodríguez, Carlos Cristi-Montero, Carlos Celis-Morales, Danica Escobar-Gómez, Palma Chillón

**Affiliations:** 1 IRyS Research Group, School of Physical Education, Pontificia Universidad Católica de Valparaíso, Valparaíso 2374631, Chile; carlos.cristi@pucv.cl (C.C.-M.); danica.escobar@hotmail.cl (D.E.-G.); 2 BHF Glasgow Cardiovascular Research Centre, Institute of Cardiovascular and Medical Science, University of Glasgow, Glasgow G128TA, UK; carlos.celis@glasgow.ac.uk; 3 Centro de Fisiologia y Biomecanica, Universidad Mayor, Santiago 8580000, Chile; 4 PROFITH “PROmoting FITness and Health through Physical Activity” Research Group, Department of Physical Education and Sport, Faculty of Sports Sciences, University of Granada, 18071 Granada, Spain; pchillon@ugr.es

**Keywords:** active transport, youth, physical activity, adolescent

## Abstract

Active commuting could contribute to increasing physical activity. The objective of this study was to characterise patterns of active commuting to and from schools in children and adolescents in Chile. A total of 453 Chilean children and adolescents aged between 10 and 18 years were included in this study. Data regarding modes of commuting and commuting distance was collected using a validated questionnaire. Commuting mode was classified as active commuting (walking and/or cycling) or non-active commuting (car, motorcycle and/or bus). Commuting distance expressed in kilometres was categorised into six subgroups (0 to 0.5, 0.6 to 1, 1.1 to 2, 2.1 to 3, 3.1 to 5 and >5 km). Car commuting was the main mode for children (to school 64.9%; from school 51.2%) and adolescents (to school 50.2%; from school 24.7%). Whereas public bus commuting was the main transport used by adolescents to return from school. Only 11.0% and 24.8% of children and adolescents, respectively, walk to school. The proportion of children and adolescents who engage in active commuting was lower in those covering longer distances compared to a short distance. Adolescents walked to and from school more frequently than children. These findings show that non-active commuting was the most common mode of transport and that journey distances may influence commuting modes in children and adolescents.

## 1. Introduction

During the last decade, physical activity levels have decreased [1] whilst sedentary-related behaviours have increased [2,3] in the Chilean adult population. These changes have been attributable to an increase in the use of motor vehicles, which drastically influence compliance with global recommendations for physical activity [4]. However, it is not only adults who have reduced their physical activity levels. Currently, 69.1% of children and adolescents do not meet the physical activity recommendations and therefore do less than one hour of moderate to vigorous physical activity (MVPA) per day [5].

Active commuting, especially in children and adolescents, has been suggested as a feasible way of increasing their overall physical levels [6]. Walking and cycling are modes of active commuting which provide numerous health benefits [7,8]. Public transport systems such as buses or trains also promote active commuting when compared to private transport (car commuting), as they are characterised by multimodal (active/non-active) transports [9]. Since commuting to and from school is conducted on a regular basis, the promotion of active commuting may be a feasible way of integrating physical activity into daily life and thus increasing the number of children and adolescents that meet the current physical activity guidelines [10].

Although there is increasing evidence regarding active commuting behaviours in children and adolescents, most of this evidence has been generated from developed countries, such as Canada, U.S., Australia and European countries [10,11,12,13,14,15,16]. However, there is a lack of evidence from developing countries, including Chile. Considering the rapid economic growth and fast transition towards a westernised lifestyle that Chile has experienced during the last three decades [6], it is essential to characterise and quantify the trend and patterns of active commuting to and from the school in children and adolescents. This information could inform public health authorities for the implementation and promotion of active commuting as a vehicle for tackling childhood obesity but also non-communicable diseases associated with a lack of physical activity [17]. The aim of this study was, therefore, to characterise patterns of active commuting to and from schools in children and adolescents in Chile.

## 2. Material and Methods

### 2.1. Study Design and Participants

This cross-sectional study was conducted in the urban city of Valparaiso in Chile. The sample is non-probabilistic and intentional. A total of 421 students (168 children and 253 adolescents) aged between 10 and 18 years old (equivalent to children in 5th and 7th grade and adolescents attending grades 8 to 12) were included in this study. These participants belonged to three public and two private schools from the Valparaíso region.

### 2.2. Instruments

The mode of commuting to and from school was measured using a questionnaire for children and adolescents developed by the University of Granada (Spain) through the project “PACO: pedalea y anda al colegio” (http://profith.ugr.es/paco). This questionnaire has been validated in Spanish population [18] and was the result of a systematic review of 158 studies in the scientific literature around the assessment of the mode of commuting using questionnaires [19]. Questions were selected based on their appropriateness towards the objective of the study. Students completed this questionnaire with assistance from their teacher. The questionaire included closed-ended questions with multiple answers. The usual mode of commuting to and from school was categorised into “active” commuting (walking and cycling) and non-active commuting (car, motorcycle, or bus). For commuting distance from home to their school participants could choose any of the following options: 0 to 0.5 km, 0.6 to 1 km, 1.1 to 2 km, 2.1 to 3 km, 3.1 to 5 km and >5 km. The reliability of the questionaire was measured in a sub-sample of 219 Chilean children and adolescents by applying the same questionnaire on two different occasions 7 days apart. The usual mode to and from school shows a high reliability (Kappa > 0.85), whereas commuting distance shows a moderate reliability (Kappa > 0.69).

### 2.3. Procedure

The questionnaire was completed between April and June 2017. The students completed the questionnaires in 15 to 30 min with the assistance of their school teachers.

### 2.4. Ethical Aspects

Prior to data collection, parents and students were informed about the characteristics of the questionnaire, the study purpose, and the confidentiality of the results. All questionnaires were answered voluntarily and with parental consent. Therefore, all participants gave informed consent for participating in the study, following the rules, with the study approved by Ethics Committee of the Pontificia Universidad Católica de Valparaíso (code: CCF02052017) and the 2004 Helsinky statement.

### 2.5. Data Analysis

The results are presented as means and standard deviations for continuous variables and as a prevalence rate for categorical variables. For analysis purposes, the sample was stratified into groups by sex and age for both children and adolescents. Differences in commuting mode between gender (boys vs. girls), age (children vs. adolescents) and commuting route (to school vs. from school) were assessed using the Chi-square test. Differences for commuting distance by age and commuting mode were also investigated using Chi-square test. All statistical analyses were performed using the statistical program SPSS^®^ version 21 (IBM, New York, NY, USA). A *p*-value (<0.05) was set as significant in all analyses.

## 3. Results

Table 1 presents the characteristics of the school children participating in the study including gender, age and type of school (Private or Public).

The modes of commuting to and from school for both children and adolescents are shown in Table 2. The proportion of children and adolescents who reported walking to and from the school was 11% and 24.8%, respectively. In children, car commuting was the main commuting mode reported (64.9% going to school and 51.2% returning from school), whereas for adolescents 50.2% reported commuting to school by car but 37.5% reported using the bus to return from school.

The modes of commuting between children and adolescents were statistically different, except for the metro or train commuting. Adolescents reported walking more frequently than children (*p* < 0.001). They also reported a lower use of car commuting and a higher use of public transport when compared to children. Interestingly, children commuted more by motorcycle (in the company of their parents) than adolescents (*p* < 0.001), while neither children nor adolescents used bicycles as a prominent mode of commuting to school.

**Figure 1:**
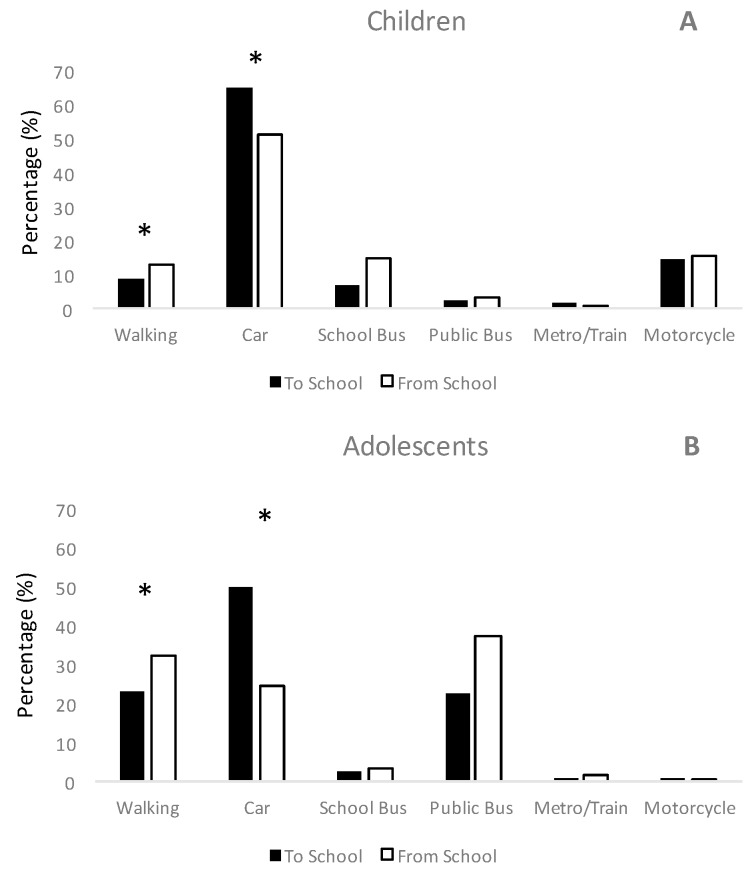
Comparison of the modes of commuting to and from school, in children (**A**) and adolescents (**B**). * *p* < 0.05 value between “To school” and “From school” in children and adolescents by car and walking.

Differences between the modes of commuting by route of commuting (to and from school) are shown in Figure 1. Car commuting was the main commuting mode used for both children and adolescents to travel from home to the school. However, car, public bus and walking were the main commuting modes used to return from school in adolescents. Car commuting remains the main commuting mode for returning from school in children.

The commuting distances from home to school in children and adolescents are shown in Table 3. Commuting distances ranging from 0 to 5 km did not show any significant differences between children and adolescents. However, commuting distances greater than 5 km are significantly higher for adolescents than for children.

**Figure 2:**
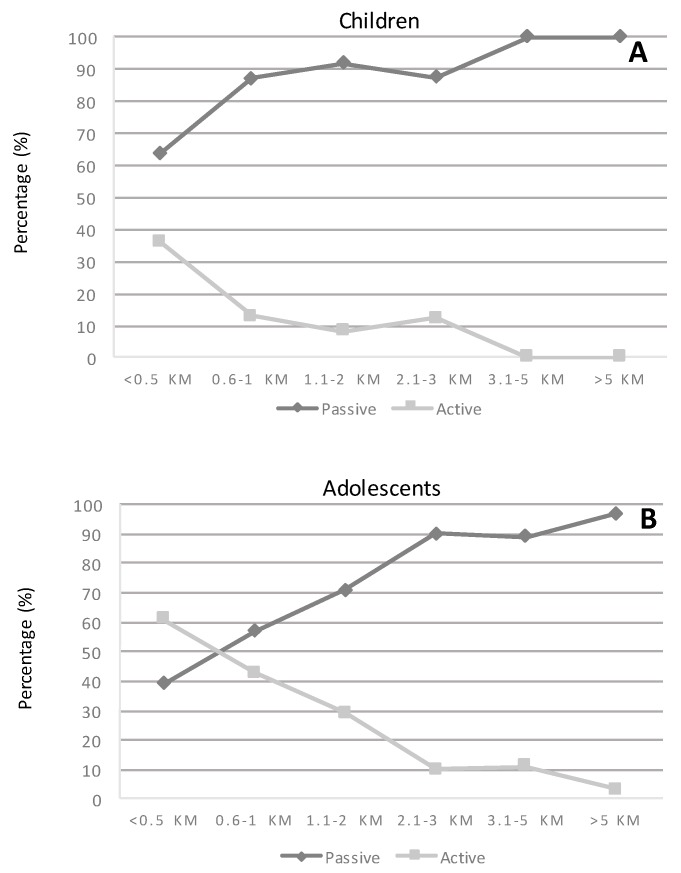
Distances from home to school according to active or non-active modes of commuting in children (**A**) and adolescents (**B**).

Figure 2 shows the percentage of children by commuting distance and commuting mode. The results show that active commuters are more likely to commute shorter distances (<1 km), whereas the proportion of non-active commuters increases for middle and long distances (>1 km) in both children and adolescents.

## 4. Discussion

The main findings of this study indicate that car commuting is the most popular commuting mode in both children (to school 64.9%; from school 51.2%) and adolescents (to school 50.2%; from school 24.7%). Most adolescents, however, used the public bus to return from school. Interestingly, walking was the only active mode used, with no participants reporting cycling to or from school. The proportion of walking commuters is higher in adolescents than in children. As expected, the percentage of both children and adolescents who walk to school was higher for those who commute short distances. It is worthy of note that this is the first study addressing patterns of different commuting modes in young Chilean students, which reinforces the need for more research on this subject.

### 4.1. Patterns of Mode of Commuting

Less than 10% of children and around 25% of adolescents reported commuting by walking in the present study. These results are lower than those reported by other developed countries such as Spain where 62.4% of schoolchildren walked or cycled to school [17], or Belgium where 65.2% of girls and 65.5% of boys used active commuting to school [16]. However, adolescents from North America showed a similar proportion of active commuting to those observed in our study [20,21], 15% of 13-year-old Canadian students walked to school [19], and only 8% of adolescents between the ages of 14 and 17 in the USA walked to school once a week [21].

Interestingly, none of the participants included in this study reported cycling to or from school. Similarly, in Spain, only 0.5% of students cycle to school [17]. However, in Belgium, 44.4% of students used a bicycle as a means of transport [16]. In the Netherlands, 31.8% of school children cycle to school [22]. Cultural and road safety differences within these countries could explain the differences observed within Chile, Belgium and The Netherlands. These countries have a large tradition of cycling, not only in students, but in the whole population [14,22]. In addition, it is important to bear in mind the geographical context of the region of Valparaíso, where the Chilean participants came from. Valparaíso has important inconveniences such as long distances from home to school and the different geography between the coast and the hills inland. Moreover, the scarce availability of bicycle paths (only 2 km) does not favour the use of this mode of transport. Other cities in Chile, such as Rancagua, Curicó, and Talca, show a higher use of bicycles in daily commuting (4%, 12% and 8%, respectively) [23]. These cities are located on flat landscapes and are characterised by being geographically more accessible.

Our results show that car commuting is the most popular mode of commuting for both children and adolescents. However, adolescents reported using public bus as the second most common mode of commuting from school to home. These data show the increasing level of independent mobility that adolescents have when compared to children. In addition, the use of public transport also implies some form of active commuting [24,25], and it is more highly recommended for young people than private transport from a health and autonomous point of view. A study conducted in the US showed that children undertake an average of 19 min physical activity to walk from home or school to the public transport stops or stations [8].

### 4.2. Distance from Home to School

This study revealed that more than 35% of children and adolescents live more than 5 km away from school and only 20% of children live less than 0.5 km away. These findings may suggest that long distances between home and school is one of the main limitations in the promotion of active commuting behaviours in children and adolescents. An Australian study [26] reported that the greater the distance between home and school, the lower the percentage of school children who actively commute every day. In addition, this study pointed out that school children who live less than 750 m away from school are more active. This data coincides with the adolescent group in the present study. It has been established that school children living less than 800 m from school, are 5 times more likely to walk [27]. A Spanish study identified 800 m as a walkable distance for children going to school [28]. In addition, other researchers established differences with cut off points at 875 m in children and 1.35 km in adolescents [29]. This means that the threshold distance for active commuting increases with age. For example, Chillón et al. [30], established a threshold distance among English young people at 1.4, 1.6 and 3.0 km for students aged 10, 11 and 14 years old, respectively.

Given that schools are far from residential areas, developing catchment areas policies to reduce distances and promote active commuting could bring the Chilean context closer to that of other countries in Europe. These results provide several insights that should be further analysed and improved on within the Chilean society. The main issue is that parents in Chile travel more than an hour on a daily basis to take their children to private schools—which are considered to be more qualified than the public schools located closer to family homes. This trend negatively affects the promotion of active commuting to school, since longer distances often force families to use their cars. It would be helpful to promote active commuting by improving governmental policies that ensure safety and reduce barriers to active commuting. Furthermore, it may help society to tackle the high inactivity and alarming childhood and adolescent obesity rate in Chile.

### 4.3. Strengths and Limitations

The main strengths of this study are that this is the first study to describe the modes of commuting in Chilean children and adolescents, and that it provides reliable data originating from the questionnaire. Limitations are the low number of total participants, the low heterogeneity of the Chilean cities included in the sample, and the self-reported distance from home to school.

## 5. Conclusions

The most common mode of commuting in children and adolescents from Valparaiso is the car, and the rate of walking was lower than in other European and North American countries. This study shows higher independent mobility in adolescents (walking and public transportation) when compared to children. In addition, the long distance from home to school appears to be a main barrier in the promotion of active modes of commuting among the Chilean school children. These results provide unique information on active commuting patterns in school children which could be used to inform public health authorities for the promotion and facilitation of active commuting in children and adolescents. This, in turn, could have important implications for health during adulthood.

## Figures and Tables

**Table 1:** Descriptive sociodemographic data of the participants.

Characteristics	Overall		Children		Adolescents	
	*n*	(%)	*n*	(%)	*n*	(%)
All	453	(100)	171	(37.7)	282	(60.6)
Gender						
Male	228	(50.3)	86	(50.3)	142	(50.4)
Female	225	(49.7)	85	(49.7)	140	(49.6)
Type of school						
Private School	258	(57.0)	85	(49.7)	173	(61.3)
Public School	195	(43.0)	91	(53.2)	104	(36.9)
Age (mean + SD)	12.7	±2.0	10.6	±0.6	13,9	±1.5

SD: Standard deviation.

**Table 2:** Mode of commuting to and from school by sex in children and adolescents.

	**Children**			**Adolescents**			
**To School**	**Overall**	**Boys**	**Girls**	**Overall**	**Boys**	**Girls**	
	***n* (%)**	***n* (%)**	***n* (%)**	***n* (%)**	***n* (%)**	***n* (%)**	***p*-Value**
Active commuting							
Walking	15 (8.9)	9 (10.7)	6 (7.1)	59 (23.3)	31 (25.4)	28 (21.4)	<0.001
Bicycle	0 (0.0)	0 (0.0)	0 (0.0)	0 (0.0)	0 (0.0)	0 (0.0)	-
Non-active commuting							
Car	109 (64.9)	54 (64.3)	55 (65.5)	127 (50.2)	54 (44.3)	73 (55.7)	0.003
School Bus	12 (7.1)	8 (9.5)	4 (4.8)	6 (2.4)	4 (3.3)	2 (1.5)	<0.001
Public Bus	4 (2.4)	3 (3.6)	1 (1.2)	57 (22.5)	33 (27.0)	24 (18.3)	0.018
Metro/Train	3 (1.8)	2 (2.4)	1.2 (1)	2 (0.8)	0 (0.0)	2 (1.5)	0.356
Motorcycle	25 (14.9)	8 (9.5)	17 (20.2)	2 (0.8)	0 (0.0)	2 (1.5)	<0.001
	**Children**			**Adolescents**			
**From School**	**Overall**	**Boys**	**Girls**	**Overall**	**Boys**	**Girls**	
	***n* (%)**	***n* (%)**	***n* (%)**	***n* (%)**	***n* (%)**	***n* (%)**	***p*-Value**
Active commuting							
Walking	23 (13.1)	14 (15.7)	9 (10.7)	81 (32.2)	44 (36.4)	37 (28.5)	<0.001
Bicycle	0 (0.0)	0 (0.0)	0 (0.0)	0 (0.0)	0 (0.0)	0 (0.0)	-
Non-active commuting							
Car	86 (51.2)	45 (53.6)	41 (48.8)	62 (24.7)	28 (23.1)	34 (26.2)	<0.001
School Bus	25 (14.9)	9 (10.7)	16 (19.0)	8 (3.2)	1 (0.8)	7 (5.4)	<0.001
Public Bus	6 (3.6)	4 (4.8)	2 (2.4)	94 (37.5)	48 (39.7)	46 (35.4)	<0.001
Metro/Train	2 (1.2)	2 (2.4)	0 (0.0)	4 (1.6)	0 (0.0)	4 (3.1)	0.734
Motorcycle	26 (15.5)	10 (11.9)	16 (19.0)	1 (0.4)	0 (0.0)	1 (0.8)	<0.001

Chi square test (*p* < 0.05) was used to assess diferecnes in commuting modes by children and adolescents. Significant differences were set at *p* < 0.05.

**Table 3:** Distances from home to school in children and adolescents.

Distance	Children		Adolescents		
	%	(*n*)	%	(*n*)	*p*-Value
0–0.5 km	13.1%	(22)	16.1%	(41)	0.711
0.6–1 km	13.7%	(23)	14.5%	(37)	0.056
1.1–2 km	14.3%	(24)	13.3%	(34)	0.078
2.1–3 km	9.5%	(16)	8.2%	(21)	0.211
3.1–5 km	13.1%	(22)	10.6%	(27)	0.621
>5 km	36.3%	(61)	37.3%	(95)	0.039

Data presented as % (number of individuals). Significant differences were accepted at *p* < 0.05.
